# Occupational class differences in suicide: evidence of changes over time and during the global financial crisis in Australia

**DOI:** 10.1186/s12888-015-0608-5

**Published:** 2015-09-21

**Authors:** Alison J. Milner, Heather Niven, Anthony D. LaMontagne

**Affiliations:** Work, Health, & Welbeing Unit, Population Health Strategic Research Centre, School of Health & Social Development, Deakin University, Building BC3.213, Burwood, Melbourne, VIC 3125 Australia; McCaughey VicHealth Centre for Community Wellbeing, Melbourne School of Population and Global Health, University of Melbourne, Melbourne, VIC Australia

**Keywords:** Occupation, Suicide, Recession, Gender, Socio-economic gradient, Skill level, Intentional self-harm, Gender

## Abstract

**Background:**

Previous research showed an increase in Australian suicide rates during the Global Financial Crisis (GFC). There has been no research investigating whether suicide rates by occupational class changed during the GFC. The aim of this study was to investigate whether the GFC-associated increase in suicide rates in employed Australians may have masked changes by occupational class.

**Methods:**

Negative binomial regression models were used to investigate Rate Ratios (RRs) in suicide by occupational class. Years of the GFC (2007, 2008, 2009) were compared to the baseline years 2001–2006.

**Results:**

There were widening disparities between a number of the lower class occupations and the highest class occupations during the years 2007, 2008, and 2009 for males, but less evidence of differences for females.

**Conclusions:**

Occupational disparities in suicide rates widened over the GFC period. There is a need for programs to be responsive to economic downturns, and to prioritise the occupational groups most affected.

**Electronic supplementary material:**

The online version of this article (doi:10.1186/s12888-015-0608-5) contains supplementary material, which is available to authorized users.

## Background

There is long-standing interest in the influence of macro-economic changes on population-level suicide rates [1], most recently in relation to the Global Financial Crisis (GFC) [2–7], which occurred over the period 2007 to 2010 in high-income countries. Changes during economic downturns can be explained in part by rising unemployment, and the increased risk of suicide among the unemployed. However, shifts in the working population must also be considered in order to gain a full understanding of the impact of economic downturns on suicide rates, particularly as absolute numbers of suicides among the employed exceed those among the unemployed (despite the higher rates associated with unemployment) [7]. Macro-economic changes could also affect disparities or inequalities in rates [8, 9]. For example, in the UK there were differential increases in suicide among those in manual occupations in the past thirty years compared to those in higher skilled jobs [10]. We recently conducted a study of all suicides in Australia over 2001–2010, showing a small and transient increase in suicide risk during the GFC period in the working population alongside a larger increase among the unemployed and economically-inactive population [7]. In the present study, we extend this work to investigate whether the small increase in suicide rates among the employed may have masked larger changes in disparities by occupational class in Australia over the 2007–2009 GFC period compared to 2001–2006. We also assessed whether gender modified the association between the GFC and suicide. We hypothesised that males would be more affected by the GFC than females. The rationale for this was based on past research demonstrating that male suicide increased in response to labour market changes (e.g., unemployment) while female suicide did not [1].

## Method

### Study design

This retrospective mortality study consisted of a time-trend analysis of suicide rates by occupational class and gender.

### Data sources

The National Coroners Information System (NCIS) is a comprehensive national internet-based data system for Australian coronial cases, established in 2001. NCIS is utilised by coroners, government agencies, and researchers for identifying cases for death investigation and research, and to monitor external causes of death in Australia. Each case on NCIS routinely includes demographic factors (age, sex, employment status, and occupation where applicable), a police description of the circumstances and background of each individual case, as well as coronial findings, autopsy and toxicology reports. Data is available for a fee and after the project has received approval from the Justice Human Research Ethics Committee (JHREC).

Population data by occupational group was obtained from the Australian Bureau of Statistics for the two census periods in 2001 and 2006. This data was coded according to the Australian and New Zealand Standard Classification of Occupations (ANZSCO) [11] by 10-year age groups (15–24 years, 25–34 years, 35–44 years, 45–54 years, 55–64 years, 65–74 years) and gender.

### Occupational coding

Available information on occupation for suicide cases was coded by two researchers using the Australian and New Zealand Standard Classification of Occupations (ANZSCO) classification [11]. To be included in this study, suicide cases had to be employed at the time of death, and be of working age (15–64 years). Of the total number of eligible 8929 employed suicide cases available in the NCIS data over the period 2001–2010, 64 cases (less than 1 %) were not able to be coded due to unclear information on occupation, leaving a total of 8865 suicide cases. The analysis in this paper focuses on the first digit level of ANZSCO (the broadest classification), which represents occupational class and skill specialisation [11] across eight major groups (in the order from highest to lowest skill): 1 = managers, 2 = professionals, 3 = technicians and trades workers, 4 = community and personal service workers, 5 = clerical and administrative workers, 6 = sales workers, 7 = machinery operators and drivers, and 8 = labourers. We analysed Farmers and Farm Managers (a subgroup within ANZSCO group 1 managers) separately as this group was hard to classify into any one single group (e.g., the term ‘farmer’ in a case file could describe either a farm manager or farm labourer). More detailed information on the procedure for coding occupation, including a description of the eight major ANZSCO occupational groups, can be seen in Additional file 1.

### Suicide rate calculation

Male and female suicide rates per 100,000 persons were calculated by broad occupational category using the 2001 and 2006 population census data by ANZSCO groupings. Rates were age-standardised using the Australian standard population (2001) [12].

### Analysis

Negative binomial regression models were used to investigate Rate Ratios (RRs) in suicide by the ANZSCO major groupings, with the highest class group (Managers) as the reference. Negative binomial was chosen over Poisson regression following the identification of over-dispersion in the regression models. The GFC years (2007, 2008, 2009) were analysed separately compared to the baseline years 2001–2006 (as one group); 2010 was also included to assess possible post-GFC related changes in suicide. The regression model controlled for age (15–24 years, 25–34 years, 35–44 years, 45–54 years, 55–64 years) and gender (female, male). Variations between ANZSCO occupational groupings and the GFC period, and between ANZSCO occupational groupings and gender were tested by including interaction terms in a regression model. This regression model was compared against a model with no interaction terms. The significance of the interaction tests was assessed using both the likelihood ratio test (LRT) and by examining the significance of interaction terms in the model. If results were significant, negative binomial regression models were stratified by pre-GFC period (2001–2006) compared to GFC years (2007, 2008, 2009), and 2010 (post-GFC). Coefficients were transformed into Rate-Ratios (RRs) to aid interpretation.

### Data access, responsibility, and analysis

AM and HN had full access to all the data in the study and takes responsibility for the integrity of the data and the accuracy of the data analysis.

### Ethics committee approval

Ethics approval for this study was grated by the Victorian Department of Justice Human Research Ethics Committee and the Melbourne School of Population and Global Health Human Ethics Advisory Group.

## Results

Table 1 describes the number of suicides for males and females in each occupational group and the corresponding age-standardised suicide rates for the entire 2001 to 2010 period. For males, the highest suicide rates were among labourers, farmers, machinery operators, and technical and trade workers. For females, the highest suicide rates were in farmers, machinery operators, labourers and professionals. As can be seen in the population data, male dominated occupational groupings included technical and trade workers, professionals, machine operators, and labourers. There were a greater number of females employed in the professional worker category, sales, community service and in clerical/administrative groupings.

**Table 1:** Total number of suicides, average population size, and age-adjusted suicide rates per 100,000 persons, over the period 2001 to 2010, males and females, Australia

	ANZSCO major grouping	Suicides (total)	Population size (average)	Age adjusted suicide rates per 100,000
Males	Managers	335	488,908	7.73
	Professionals	911	776,735	13.29
	Tech/trade	2,387	1,081,134	21.12
	Comm Service	459	275,976	17.2
	Clerical admin	295	3,170,080	5.26
	Sales	361	376,916	13.59
	Machinery	985	559,941	20.83
	Labourers	1,536	562,885	34.6
	Farmers	236	130,679	19.03
Females	Managers	60	254,981	2.72
	Professionals	382	846,019	5.12
	Tech/trade	117	173,422	3.64
	Comm Service	237	510,282	3.7
	Clerical admin	258	9,888,775	1.39
	Sales	135	545,130	2.94
	Machinery	20	77,776	2.92
	Labourers	133	316,143	5.24
	Farmers	18	56,133	3.31

The main effects model with the interaction can be seen in Table 2. This suggests that males had 4-fold higher RR than females for the entire 10-years of data available. Compared to the suicide rate in managers, labourers, those employed in technical and trades, farmers and machine operators had particularly elevated RRs of suicide. The interaction tests indicated significant differences in the relationship between occupation and suicide by sex (LRT *x*2(8) = 211.56, *p* < 0.001).

**Table 2:** The main model and model with interaction terms, rate ratios with 95 % confidence intervals comparing major occupational groups to the suicide rate in managers (the highest class group), 2001 to 2010

	Main effects model			Interaction model		
	RR	95 % CI	*p* value	RR	95 % CI	*p* value
Managers	Reference			Reference		
Professionals	174	1.50, 2.01	<0.001	1.37	1.02, 1.84	0.035
Tech/trade	2.30	1.99, 2.66	<0.001	1.14	0.82, 1.59	0.433
Comm service	1.80	1.54, 2.10	<0.001	1.03	0.76, 1.40	0.867
Clerical admin	1.25	1.06, 1.46	0.007	0.81	0.60, 1.09	0.159
Sales	1.62	1.38, 1.90	<0.001	0.92	0.67, 1.23	0.634
Machinery	2.27	1.94, 2.65	<0.001	2.07	1.22, 3.51	0.007
Labourers	3.62	3.13, 4.19	<0.001	1.60	1.15, 2.21	0.005
Farmers	2.55	2.11, 3.08	<0.001	2.32	1.34, 4.00	0.003
Year	0.96	0.90, 1.11	0.145	0.95	0.89, 1.01	0.076
Female	Reference			Reference		
Male	3.87	3.58, 4.17	<0.001	2.19	1.62,2.94	<0.001
15–24 years	Reference			Reference		
25–34 years	1.60	1.44, 1.77	<0.001	1.59	1.43, 1.76	<0.001
35–44 years	1.63	1.47, 1.80	<0.001	1.64	1.48, 1.81	<0.001
45–54 years	1.53	1.38, 1.70	<0.001	1.55	1.40, 1.71	<0.001
55–64 years	1.29	1.15, 1.45	<0.001	1.29	1.16, 1.45	<0.001
Managers*Females				Reference		
Prof*Males				1.22	0.88, 1.71	0.237
Tech/trade*Males				2.31	1.61, 3.33	<0.001
Comm Service*Males				2.05	1.45, 2.91	<0.001
Clerical admin*Males				1.67	1.17, 2.37	0.581
Sales*Males				2.02	1.40, 2.92	<0.001
Machinery*Males				1.20	0.69, 2.07	0.523
Labourers*Males				2.73	1.90, 3.90	<0.001
Farmers*Males				1.18	0.66, 2.10	0.581

Notes: 95 % *CIs* 95 % Confidence intervals (lower, upper); *RR* rate ratios; *p* value = significance value 95 %

**Fig. 1:**
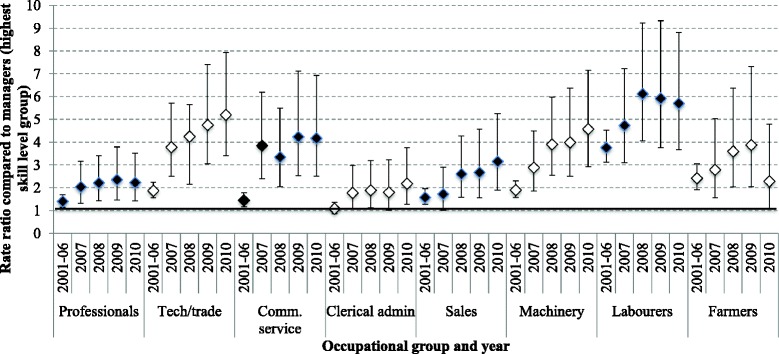
Rate ratios with 95 % confidence intervals comparing major occupational groups to the suicide rate in managers (the highest level group, indicated by 1 on the left hand side axis), males. Notes: Upper and lower bars = 95 % Confidence intervals (*lower*, *upper*); dot points = Rate ratios; Reference category (1 on *vertical* axis) = suicide rate in managers (2001–2006 = 5.83 per 100,000; 2007 = 3.24 per 100,000; 2008 = 2.20 per 100,000; 2009 = 2.50 per 100,000; 2010 = 2.34 per 100,000)

Results of the interaction test examining variation in RRs in the ANZSCO occupational groupings (with managers as the reference group) by time period (i.e. GFC years 2007, 2008, 2009 versus pre-GFC period 2001–2006) and by sex, were also significant (LRT *x*2(40) = 162.12, *p* < 0.001). This suggests that the differences in RRs of lower skilled occupations compared to higher skilled varied over the GFC period compared to pre-GFC, and that this relationship differed for males and females. Based on these results, models were stratified by time period and by sex. Figure 1 shows the stratified results for males (these results are also available in Additional file 2). The horizontal line marked in bold represents suicide among managers, which is the reference category for each occupational group. For males, the ratio of suicide in managers (the reference category) to professionals, technical and trade workers, community service workers, sales workers, machinery operators, labourers and farmers increased during the GFC and remained high in the year 2010 (the rate in farmers decreased but remained above managers in 2010). The rate of suicide in managers dropped slightly over the period from 5.83 per 100,000 to 2.20 per 100,000 (see footnote at the bottom of Fig. 1).

There was also a three-fold increase in the disparity of suicide rates in technical and trade workers and community service workers. Compared to managers, the RR among technical and trade workers in 2001–2006 (RR 1.87, 95 % CI 1.56 to 2.24, *p* < 0.001) rose to 4.25 (95 % CI 2.84 to 6.35, *p* < 0.001) in 2008 and continued to increase into the year 2010 (RR 5.20, 95 % CI 3.41 to 7.92, *p* < 0.001). Community service workers had an RR of 1.45 (95 % CI 1.17, 1.79, *p* = 0.001) compared to managers pre-GFC. This RR increased to 3.85 in 2007 (95 % CI 2.39 to 6.19, *p* < 0.001) and continued to increase into 2010 (RR 4.17, 95 % CI 2.51 to 6.92, *p* < 0.001). Although not being significantly higher, the 2001–2006 RR for male labourers compared to managers nearly doubled during 2008 (RR 6.12; 95 % CI 4.06 to 9.22, *p* < 0.001) and 2009 (RR 5.91; 95 % CI 3.76 to 9.32, *p* < 0.001), with only a slight decline in 2010 (RR 5.70; 95 % CI 3.68 to 8.82, *p* < 0.001).

**Fig. 2:**
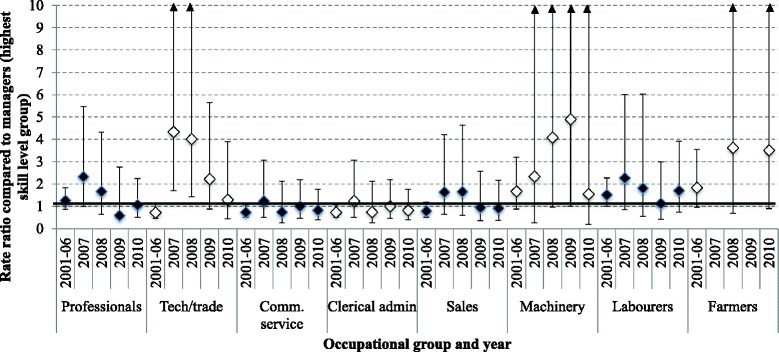
Rate ratios with 95 % confidence intervals comparing major occupational groups to the suicide rate in managers (the highest level group, indicated by 1 on the left hand side axis), females. Notes: Upper and lower bars = 95 % Confidence intervals (*lower*, *upper*); dot points = Rate ratios; Reference category (1 on *vertical* axis) = suicide rate in managers. The *arrows* on tech/trade represent 95 % upper CIs that are larger than 10. (2001–2006 = 2.11 per 100,000; 2007 = 1.11 per 100,000; 2008 = 1.23 per 100,000; 2009 = 1.69 per 100,000; 2010 = 1.21 per 100,000)

Figure 2 shows stratified results for females (see Additional file 3 for results in tabular format) and the rate of suicide in managers, which dropped from 2.11 per 100,000 to 1.11 per 100,000. Due to a low count of suicides in some occupational groups, a number of estimates were unstable which hinders interpretation of results. There was no significant elevation for technical and trade workers compared to managers during the pre-GFC period of 2001–2006. However, there was a four-fold increase in suicide among technical and trade workers compared to managers in 2007 (RR 4.33, 95 % CI 1.71, 10.98, *p* = 0.002) and 2008 (RR 4.01, 95 % CI 1.42, 11.35, *p* = 0.009) and a non-significant decline in 2009 and 2010. There was also an increase among females employed as machinery workers compared to managers during the GFC. The RR was 1.67 in 2001–2006 (95 % CI 0.88, 3.19, *p* = 0.120) and increased to 4.90 in 2009 (95 % CI 1.02, 23.52, *p* = 0.047). The RRs for technical and trade workers and machine operators declined in 2010.

## Discussion

### Main findings

Over the period 2001 to 2010, major occupational groups with the highest rates of suicide in Australia were labourers, farmers, machine operators and technical and trade workers. There was some evidence of gender differences in rates of suicide, although both males and females had higher rates in labouring jobs. The global financial crisis was associated with increasing class disparities in suicide rates. This was most apparent among males in manual occupations.

### Occupational groups at risk of suicide over the period 2001 to 2010

A number of studies have documented an elevated risk of suicide among unskilled labouring workers [7, 8, 10, 13–15]. Higher suicide rates among females and males employed in these occupations have been attributed to a range of factors including socio-economic disadvantage [10] and exposure to stressful working conditions such as low job control and high demands [7]. The results of the present study also suggest that males generally had higher suicide rates in occupations involving physical work, including labouring, agriculture, machine operators, and technical and trades employment. This finding aligns with past research on suicide among males employed in the construction [16, 17] and agricultural industries [9]. For females, an elevated rate of suicide was found for the professional occupational group (ANZSCO group 2), which includes doctors and other medical professionals such as nurses. Suicide among those employed in medical jobs has been attributed to greater access to means (prescription medications), job pressures, and underlying psychological vulnerabilities and mental health issues [18, 19]. It may be that these risk factors affect females more than males, leading to their higher suicide rate. However, we should also note small sample size for females and the decreasing rate of suicide among managers. This reflects a general downward trend in the overall national suicide rates over this period, further emphasising the difference from other occupational groups. There were a number of other differences between male and female suicide over time, such as the declining rate of suicide among females in technical and trades occupations, while their male counterparts had increasing suicide. However, the small number of suicides occurring in females means that these results were unreliable. This highlights the need for greater need for research into gender difference in suicide by occupation. We would suggest a qualitative approach to this topic may be most informative in unpacking these relationships.

### The association between the GFC and disparities in suicide rate by occupational class

The GFC in Australia coincided with a widening of disparities in suicide rates by occupational class, particularly among males. Suicide rates in unskilled workers such as labourers increased from three to six-fold greater than the rates in the highest class group, while there was over a four-fold increase among technical and trade workers. Disparities persisted into the year 2010, which suggests that higher rates among unskilled labourers, and technical and trade workers, was a continuing problem. These findings correspond with research from Korea regarding the economic recession in the late 1990s, which produced persistent and widening differences in depression, suicide ideation and suicide attempts [19]. The Korean study found that the lowest income group was consistently disadvantaged compared to higher income groups [19]. Similarly, a study on suicide in the construction industry in Australia found that labourers had significantly elevated rates during the GFC compared to higher skilled occupations [17].

There are a number of potential explanations for widening occupational disparities in suicide rates. First of all, this may be connected to increasing social disparities and economic disadvantages experienced during the GFC [10, 20, 21]. It may also be related to changes in social and economic welfare policies [2, 5, 21] and rising job insecurity [22], and more precarious employment, each of which would be more likely to affect those in lower skilled jobs. Certainly, there is evidence to suggest that lower skilled workers report greater job insecurity more than higher skilled workers [22] and that job insecurity rose in Australia during 2008 with the onset of the GFC [23]. Although there is a lack of research specifically on suicide, job insecurity predicts common mental disorders [24]. Further, lower skilled workers experience considerably higher unemployment rates than many other occupational groups in Australia [25]. In fact, the number of labourers who became unemployed in 2013 were over three times the number of unemployed in managerial or professional occupations [26]. Hence, lower skilled workers may be disproportionally concerned about their future employment prospects (even while in paid work). Another possible explanation is shifting of persons in higher class jobs into lower class jobs (demotion).

### Limitations

The limitations of this study include under-reporting of suicide. This was particularly problematic in the initial years during which NCIS was established [27, 28] but may be an ongoing problem. Other possible problems include the miscoding of occupation, which may have occurred despite independent coding by two researchers and the use of a structured approach to the classification. We also acknowledge that we have used a rather crude measure of the GFC. The enormity of global events such as the most recent economic downturn means that different groups of people may have been vulnerable at different times and that the duration and intensity of the GFC could have varied by occupational group [29]. Our “before/after” year measure would have been unable to distinguish these differences. Further, small numbers of suicides in some occupational groups meant that we were unable to assess statistical significance. Notwithstanding these issues, this study has a number of strengths, including its use of the best available quality population-level data on suicide, and coverage across an entire national population over a ten year period.

## Conclusion

This paper demonstrated that occupational disparities in suicide rates are dynamic over time, and can be exacerbated by economic downturns such as the 2007–2009 GFC. Further, the cessation of the GFC by 2010 was not associated with a marked reduction in rates of suicide in many occupational groups, which could indicate lasting impacts. From a suicide prevention perspective, a wide range of policy interventions from the individual support program to macro-economic policy levels should be considered, tailored in particular to the occupational groups most at risk of suicide during economic downturns.

## Additional files

Additional file 1:
**Information on the coding of occupation.** (DOC 25 kb) Additional file 2:
**Rate ratios with 95 % confidence intervals comparing major occupational groups to the suicide rate in managers (the highest skill level group), males.** (DOC 40 kb) Additional file 3:
**Rate ratios with 95 % confidence intervals comparing major occupational groups to the suicide rate in managers (the highest skill level group), females.** (DOC 44 kb)

## Abbreviations

ANZSCO: Australian and New Zealand Standard Classification of Occupations
GFC: Global financial crisis
RR: Rate ratios
JHREC: Justice Human Research Ethics Committee
NCIS: National Coroners Information System
