# Supplementary material for: Occupational class differences in suicide: evidence of changes over time and during the global financial crisis in Australia
Source: BMC Psychiatry. 2015 Sep 21;15:223. doi: 10.1186/s12888-015-0608-5 (PMC4578370; doi:10.1186/s12888-015-0608-5)
Supplement: Additional file 1: — Information on the coding of occupation. (DOC 25 kb) [file 12888_2015_608_MOESM1_ESM.doc]

**Additional File 1: Information on the coding of occupation**

When coding occupational information, if more than one occupation was reported, the researchers took the first listed as the primary occupation, unless the second listed occupation provided additional information that the first could not offer (e.g., construction/carpenter). Ambiguous occupational information was coded at the broadest level where possible, or was marked as uncodable. Uncodable occupational data were either unclear (such as “chief”) or too broad to be coded even to the major eight-group level, such as apprentice, self-employed, controller, supervisor and team leader, where no other qualifying information was provided. However, when more specific information was present, such as “apprentice mechanic”, this was able to be coded. Since the ANZSCO classification does not code specifically for apprentices, these have been coded as the occupation they are training in (i.e. “apprentice mechanic” has been coded as “mechanic”). Consensus was reached via discussion between researchers, and in some cases experts in certain industries (such as construction and engineering) were consulted when it was deemed useful to clarify industry-specific occupational data. The database was also checked for consistency in coding, duplicates and other coding errors.

**ANZSCO Major groupings**

**Group 1: Managers:** plan, organise, direct, control, coordinate and review the operations of government, commercial, agricultural, industrial, non-profit and other organisations, and departments. They are usually the head of an organisation or of a specific department, and are in charge of hiring and firing of staff and responsible for planning business activities to reach company targets.

**Group 2: Professionals**: perform analytical, conceptual and creative tasks through the application of theoretical knowledge and experience in the fields of the arts, media, business, design, engineering, the physical and life sciences, transport, education, health, information and communication technology, the law, social sciences and social welfare.

**Group 3: Technicians and trades workers:** perform a variety of skilled tasks, applying broad or in-depth technical, trade or industry specific knowledge, often in support of scientific, engineering, building and manufacturing activities.

**Group 4: Community and personal service workers**: assist Health Professionals in the provision of patient care, provide information and support on a range of social welfare matters, and provide other services in the areas of aged care and childcare, education support, hospitality, defence, policing and emergency services, security, travel and tourism, fitness, sports and personal services.

**Group 5: Clerical and administrative workers:** provide support to Managers, Professionals and organisations by organising, storing, manipulating and retrieving information.

**Group 6: Sales workers:** sell goods, services and property, and provide sales support in areas such as operating cash registers and displaying and demonstrating goods.

**Group 7: Machinery operators and drivers:** operate machines, plant, vehicles and other equipment to perform a range of agricultural, manufacturing and construction functions, move materials, and transport passengers and freight.

**Group 8: Labourers:** perform a variety of routine and repetitive physical tasks using hand and power tools, and machines either as an individual or as part of a team assisting more skilled workers such as Trades Workers, and Machinery Operators and Drivers.
