# Supplementary material for: Occupational class differences in suicide: evidence of changes over time and during the global financial crisis in Australia
Source: BMC Psychiatry. 2015 Sep 21;15:223. doi: 10.1186/s12888-015-0608-5 (PMC4578370; doi:10.1186/s12888-015-0608-5)
Supplement: Additional file 2: — Rate ratios with 95 % confidence intervals comparing major occupational groups to the suicide rate in managers (the highest skill level group), males. (DOC 40 kb) [file 12888_2015_608_MOESM2_ESM.doc]

**Additional File 2. Rate ratios with 95% confidence intervals comparing major occupational groups to the suicide rate in managers (the highest skill level group), males.**

| **ANZSCO major**  **grouping 1** | **RR 2001-06** | **95% CIs** | **p value** | **RR 2007** | **95% CIs** | **p value** | **RR 2008** | **95% CIs** | **p value** | **RR 2009** | **95% CIs** | **p value** | **RR 2010** | **95% CIs** | **p value** |
| --- | --- | --- | --- | --- | --- | --- | --- | --- | --- | --- | --- | --- | --- | --- | --- |
| **Managers** | reference | |  | reference | | | Reference | | | reference | | | reference | | |
| **Profess- ionals** | 1·39 | *1·15, 1·70* | <0·001 | 2·04 | *1·31, 3·16* | =0·001 | 2·21 | *1·44, 3·41* | <0·001 | 2·35 | *1·46, 3·79* | <0·001 | 2·22 | *1·41, 3·51* | <0·001 |
| **Tech/trade** | 1·87 | *1·56, 2·24* | <0·001 | 3·78 | *2·50, 5·70* | <0·001 | 4·24 | *2·84, 6·34* | <0·001 | 4·75 | *3·05, 7·39* | <0·001 | 5·19 | *3·41, 7·92* | <0·001 |
| **Com Service** | 1·44 | *1·17, 1·77* | <0·001 | 3·85 | *2·39, 6·19* | <0·001 | 3·34 | *2·04, 5·48* | <0·001 | 4·23 | *2·52, 7·11* | <0·001 | 4·17 | *2·51, 6·92* | <0·001 |
| **Clerical admin** | 1·09 | *0·86, 1·36* | 0·489 | 1·77 | *1·05, 2·99* | 0·032 | 1·89 | *1·12, 3·19* | 0·018 | 1·79 | *1·01, 3·21* | 0·048 | 2·18 | *1·26, 3·76* | 0·005 |
| **Sales** | 1·57 | *1·26, 1·96* | <0·001 | 1·72 | *1·02, 2·91* | 0·044 | 2·60 | *1·58, 4·27* | <0·001 | 2·67 | *1·56, 4·57* | <0·001 | 3·16 | *1·89, 5·26* | <0·001 |
| **Machinery** | 1·99 | *1·57, 2·30* | <0·001 | 2·89 | *1·85, 4·49* | <0·001 | 3·90 | *2·55, 5·97* | <0·001 | 3·99 | *2·49, 6·38* | <0·001 | 4·57 | *2·93, 7·14* | <0·001 |
| **Labourers** | 3·76 | *3·12, 4·53* | <0·001 | 4·73 | *3·10, 7·21* | <0·001 | 6·12 | *4·06, 9·22* | <0·001 | 5·92 | *3·76, 9·32* | <0·001 | 5·70 | *3·68, 8·82* | <0·001 |
| **Farmers** | 2·42 | *1·91, 3·05* | <0·001 | 2·79 | *1·55, 5·03* | =0·001 | 3·59 | *2·03, 6·37* | <0·001 | 3·88 | *2·05, 7·32* | <0·001 | 2·28 | *1·09, 4·79* | 0·029 |

Notes: 95% CIs = 95% Confidence intervals (lower, upper); RR= Rate ratios; p value= significance value 95%
