# Supplementary material for: Occupational class differences in suicide: evidence of changes over time and during the global financial crisis in Australia
Source: BMC Psychiatry. 2015 Sep 21;15:223. doi: 10.1186/s12888-015-0608-5 (PMC4578370; doi:10.1186/s12888-015-0608-5)
Supplement: Additional file 3: — Rate ratios with 95 % confidence intervals comparing major occupational groups to the suicide rate in managers (the highest skill level group), females. (DOC 44 kb) [file 12888_2015_608_MOESM3_ESM.doc]

**Additional File 3. Rate ratios with 95% confidence intervals comparing major occupational groups to the suicide rate in managers (the highest skill level group), females.**

| **ANZSCO major**  **grouping 1** | **RR 2001-06** | **95% CIs** | **p value** | **RR 2007** | **95% CIs** | **p value** | **RR 2008** | **95% CIs** | **p value** | **RR 2009** | **95% CIs** | **p value** | **RR 2010** | **95% CIs** | **p value** |
| --- | --- | --- | --- | --- | --- | --- | --- | --- | --- | --- | --- | --- | --- | --- | --- |
| **Managers** | reference | | | reference | | | Reference | | | reference | | | Reference | | |
| **Profess- ionals** | 1·26 | *0·87, 1·82* | 0·224 | 2·33 | *0·99, 5·47* | 0·052 | 1·67 | *0·64, 4·32* | 0·293 | 1·27 | *0·59, 2·76* | 0·538 | 1·06 | *0·50, 2·24* | 0·879 |
| **Tech/trade** | 0·72 | *0·47, 1·09* | 0·128 | 4·33 | *1·71, 10·98* | 0·002 | 4·01 | *1·42, 11·35* | 0·009 | 2·22 | *0·88, 5·64* | 0·092 | 1·29 | *0·43, 3·90* | 0·648 |
| **Com Serv** | 0·73 | *0·50, 1·07* | 0·106 | 1·84 | *0·74, 4·58* | 0·193 | 2·08 | *0·78, 5·59* | 0·145 | 1·78 | *0·80, 3·96* | 0·157 | 1·69 | *0·78, 3·64* | 0·18 |
| **Clerical admin** | 0·73 | *0·49, 1·07* | 0·105 | 1·24 | *0·50, 3·07* | 0·646 | 0·74 | *0·26, 2·12* | 0·578 | 1·00 | *0·46, 2·20* | 0·995 | 0·82 | *0·38, 1·77* | 0·617 |
| **Sales** | 0·78 | *0·52, 1·18* | 0·260 | 1·64 | *0·64, 4·20* | 0·337 | 1·66 | *0·59, 4·64* | 0·337 | 0·94 | *0·35, 2·57* | 0·911 | 0·91 | *0·38, 2·17* | 0·83 |
| **Machinery** | 1·67 | *0·88, 3·19* | 0·120 | 2·34 | *0·27, 20·09* | 0·439 | 4·08 | *0·97, 17·10* | 0·054 | 4·90 | *1·02, 23·52* | 0·047 | 1·55 | *0·19, 12·39* | 0·68 |
| **Labourers** | 1·51 | *1·01, 2·27* | 0·045 | 2·27 | *0·86, 6·00* | 0·098 | 1·81 | *0·55, 6·03* | 0·331 | 1·13 | *0·43, 2·99* | 0·806 | 1·70 | *0·74, 3·91* | 0·208 |
| **Farmers** | 1·83 | *0·95, 3·55* | 0·070 | nil |  |  | 3·62 | *0·68, 19·17* | 0·130 | nil |  |  | 3·51 | *0·90, 13·62* | 0·070 |

Notes: 95% CIs = 95% Confidence intervals (lower, upper); RR= Rate ratios; p value= significance value 95%
